# 7,8-dihydroxyflavone, a small molecular TrkB agonist, is useful for treating various BDNF-implicated human disorders

**DOI:** 10.1186/s40035-015-0048-7

**Published:** 2016-01-06

**Authors:** Chaoyang Liu, Chi Bun Chan, Keqiang Ye

**Affiliations:** School of Information and Safety Engineering, Zhongnan University of Economics and Law, Wuhan, 430073 P.R. China; Department of Physiology, University of Oklahoma Health Sciences Center, 940 Stanton L. Young Blvd., Oklahoma City, OK 73104 USA; Department of Pathology and Laboratory Medicine, Emory University School of Medicine, 615 Michael Street, Atlanta, GA 30322 USA

**Keywords:** Flavonoids, Neurotrophin, BDNF, Mimetic compound, Receptor agonistic activity

## Abstract

Brain-derived neurotrophic factor (BDNF) regulates a variety of biological processes predominantly via binding to the transmembrane receptor tyrosine kinase TrkB. It is a potential therapeutic target in numerous neurological, mental and metabolic disorders. However, the lack of efficient means to deliver BDNF into the body imposes an insurmountable hurdle to its clinical application. To address this challenge, we initiated a cell-based drug screening to search for small molecules that act as the TrkB agonist. 7,8-Dihydroxyflavone (7,8-DHF) is our first reported small molecular TrkB agonist, which has now been extensively validated in various biochemical and cellular systems. Though binding to the extracellular domain of TrkB, 7,8-DHF triggers TrkB dimerization to induce the downstream signaling. Notably, 7,8-DHF is orally bioactive that can penetrate the brain blood barrier (BBB) to exert its neurotrophic activities in the central nervous system. Numerous reports suggest 7,8-DHF processes promising therapeutic efficacy in various animal disease models that are related to deficient BDNF signaling. In this review, we summarize our current knowledge on the binding activity and specificity, structure-activity relationship, pharmacokinetic and metabolism, and the pre-clinical efficacy of 7,8-DHF against some human diseases.

## Background

Neurotrophins (NT) are growth factors that regulate the development and maintenance of the peripheral and the central nervous systems [1]. It is a family of secretary proteins that includes brain-derived neurotrophic factor (BDNF), nerve growth factor (NGF), NT-3, and NT-4/NT-5 [2]. BDNF exerts its biological functions on neurons through two transmembrane receptors: the p75 neurotrophin receptor (p75NTR) and the TrkB receptor tyrosine kinase, while NGF binds to TrkA, NT-4/5 binds to TrkB, and NT-3 preferentially binds to TrkC [3, 4]. TrkB is one of the most widely distributed neurotrophic receptors (NTRs) in the brain, which is highly enriched in the neocortex, hippocampus, striatum, and brainstem [5]. Binding of BDNF to TrkB receptor triggers its dimerization through conformational changes and autophosphorylation of tyrosine residues in the intracellular domain, resulting in activation of signaling pathways involving mitogen-activated protein kinase (MAPK), phosphatidylinositol 3-kinase (PI3K) and phospholipase C-γ (PLC-γ).

BDNF is of particular therapeutic interest because of its neurotrophic actions on a number of neuronal populations including sensory neurons (implicated in peripheral sensory neuropathies [6]); motor neurons which are degenerated in amyotrophic lateral sclerosis (ALS) [7]; dopaminergic neurons of the substantia nigra, which are lost in Parkinson’s disease (PD); and cholinergic neurons of the basal forebrain, that play a significant role in in Alzheimer’s disease (AD) [8]. Moreover, BDNF protects hippocampal neurons from glutamate toxicity [9], rescues cerebellar neurons from programmed cell death [10], reduces ischemic neuronal injury [11, 12] and improves functional recovery from postinjury regeneration [13]. Thus, BDNF may represent a beneficial therapeutic agent against a variety of human disorders such as ALS, AD, PD, fetal alcohol exposure, autism and schizophrenia [14]. However, the outcomes of several clinical trials using recombinant BDNF are disappointing [15, 16], possibly because of the poor delivery and short in vivo half-life of BDNF. In addition, BDNF binds to p75NTR, a promiscuous NTR that is known to activate cell death pathways via recruitment of multiple adaptor proteins [17, 18]. To address these problems, tremendous effort has been made to generate selective agonists of TrkB including monoclonal antibodies [19] and peptide mimetics [20, 21] . Based on the fact that a monocyclic monomeric peptide resembling the a single loop of BDNF (loop 2) acts as an inhibitor of BDNF-mediated neuronal survival, O’Leary and Hughes designed bicyclic dimeric peptides that mimic a pair of solvent-exposed loops for the binding and activation of TrkB. These dimeric peptides behave as partial agonists of TrkB with respect to BDNF, which promotes the survival of embryonic chick sensory neurons in culture [20]. Recently, the tandem repeat peptide agonist approach has been employed for designing BDNF/NT-4/5 mimetics but none of these peptidyl compound exhibit satisfactory in vivo agonistic effect on TrkB [22]. Adenosine and pituitary adenylate cyclase-activating peptide (PACAP) has also been reported to transactivate TrkB in cultured hippocampal neurons, however, this G protein-coupled receptor ligand does not act as TrkB agonists as it only activates an immature form of TrkB after 1 h treatment [23–25].

To search for small molecules that mimic the biological functions of BDNF, we developed a cell-based TrkB receptor-dependent survival assay system, and used it to screen a chemical library via counter screening. The positive hits were then subjected to TrkB activation analysis in primary neurons and receptor binding assays. Finally, we identified two lead compounds: 7,8-dihydroxyflavone and deoxygedunin [26, 27], which bind to the receptor extracellular domain of TrkB, promote the receptor dimerization and autophosphorylation, and activate the downstream signaling cascades. Due to its commercial availability, and the favorable chemical and physical characteristics, 7,8-DHF is has been extensively explored in a variety of cell types and disease models since its first report in 2010 [26]. In the current review, we will summarize the main biochemical, physiological, pharmacological and functional activities of 7,8-DHF. Its therapeutic potentials against neurological and metabolic disorders will also be discussed.

## Discovery of TrkB receptor agonists

In order to identify small molecules that mimic the neurotrophic activities of BDNF, we developed a cell-based survival assay using a cell permeable fluorescent dye MR(DERD)2, which produces red signal upon caspase-3 cleavage in apoptotic cells. TrkB-lacking SN56 cells (a fusion of N18TG2 neuroblastoma cells with mouse neurons from postnatal day 21- septa) and its derived cell line T48 (which is stably transfected with TrkB) were utilized in our assay. We cultured the cells in 96-well plates and pre-incubated the cells with compounds from a library for 30 min, followed by staurosporine (STS) treatment for 9 h. MR(DEVD)2 was introduced to the cells 1 h before examination under fluorescent microscope. The apoptotic cells could be detected by the red signal, while the live cells had no signal. Using this caspase-activated fluorescent dye as a visual assay, we screened thousands of compounds from the Spectrum Collection library. Sixty-six compounds were found to selectively protect T48, but not SN56 cells, from STS-initiated apoptosis, indicating that these compounds might act either directly through TrkB receptor or its downstream signaling effectors. These positive hits were further analyzed on primary hippocampal neurons for TrkB activation and neuronal survival. 7,8-DHF is one of the positive compounds that specifically activate TrkB, but not TrkA or TrkC, at a concentration of 250 nM. It is also a bioavailable chemical that can pass through the BBB to provoke TrkB and its downstream PI3K/Akt and MAPK activation in mouse brain (cortex, hippocampus and hypothalamus) upon intraperitoneal or oral administration. In addition to cortical and hippocampal neurons, 7,8-DHF also protects other cell types including the RGC (retinal ganglion cells) and PC12 cells from excitotoxic and oxidative stress-induced apoptosis and cell death [28–30]. Fitting with these in vitro neuroprotective actions, 7,8-DHF protects the RGC cells from excitotoxic and oxidative stress-induced apoptosis in retinal glaucoma model in a TrkB-dependent manner [28]. Moreover, 7,8-DHF promotes the survival and reduces apoptosis in cortical neurons of TBI (traumatic brain injury) as administration of 7,8-DHF at 3 h post-injury reduces brain tissue damage via the PI3K/Akt pathway [31].

To demonstrate that 7,8-DHF indeed is a TrkB specific agonist, we performed in vitro filter binding assay with [^3^H]-labeled 7,8-DHF and purified recombinant TrkB ECD (extracellular domain) proteins. We showed that 7,8-DHF has a K_d_ of ~320 nM toward TrkB with a binding ratio of 1:1 (ligand versus receptor). Similarly, Biocore Surface Plasmon Resonance (SPR) and fluorescent quenching assay support the notion that 7,8-DHF directly binds to TrkB ECD with *Kd* about 15.4 nM. As the positive control, BDNF displays a *Kd* of approximately 1.7 nM. These assays also demonstrated that 7,8-DHF but not 5,7-DHF (5,7-dihydroxyflavone) interacts with TrkB but not TrkA [32]. The different binding affinities of 7,8-DHF towards TrkB obtained in these assays might be a result of distinctive binding principles and experimental conditions. Mapping assays further suggest that 7,8-DHF may directly interact with LRM/CC2 regions of TrkB ECD [26], a finding that is further supported by an subsequent molecular modeling and docking analysis [33]. Utilizing TrkB-Fc, a His-tagged fusion protein of human TrkB-ECD (C32-H430), and human IgG1 that can specifically neutralize BDNF’s agonistic effect on TrkB, we demonstrated that these agents can antagonize 7,8-DHF’s stimulatory effect, emphasizing that 7,8-DHF exerts its agonistic activity via binding to TrkB receptor. We tried to determine the co-crystal structure of 7,8-DHF and TrkB ECD but no satisfactory results were obtained possibly because of the highly glycosylated nature of TrkB receptor. Nevertheless, all these independent experiments strongly support that 7,8-DHF indeed preferentially interacts with TrkB but not TrkA or TrkC receptors. Since no interaction or functional studies have been performed between 7,8-DHF and the low affinity BDNF receptor p75NTR, it remains unknown if 7,8-DHF also activates p75NTR as well.

## TrkB receptor internalization by 7,8-DHF

Internalization of the neurotrophin–Trk complex plays a critical role in signal transduction that initiates cell body responses to target-derived neurotrophins. The neurotrophin–Trk complex is internalized through clathrin-mediated endocytosis, leading to the formation of signaling endosomes [34, 35]. Using two independent approaches, we demonstrated that 7,8-DHF treatment triggers an internalization of activated TrkB to the early endosomes [32]. First, we analyzed TrkB receptor endocytosis in primary neurons upon BDNF or 7,8-DHF stimulation using microscopic measurement. While internalized TrkB receptors colocalized with EEA1, the early endosome marker, kinetic quantification of the co-localized TrkB/EEA1 indicates that BDNF is more potent than 7,8-DHF in stimulating TrkB internalization and early endosomes delivery in the first 10 min. At 60 min stimulation, both BDNF and 7,8-DHF substantially increase TrkB endocytosis and its early endosomal residency but 7,8-DHF seems more robust than BDNF. Second, we performed the biotinylation assay by labeling the surface protein with sulfo-NHS-SS-biotin, followed by BDNF or 7,8-DHF stimulation. Internalized biotinylated proteins were then precipitated with streptavidin, and analyzed by immunoblotting with anti-TrkB antibody against its ECD domain. Concur with the microscopic detection, both BDNF and 7,8-DHF strongly escalate TrkB internalization.

BDNF treatment elicits TrkB receptor ubiquitination and degradation [36–39]. Our recent report found that BDNF induced TrkB ubiquitination 10 min after stimulation [32]. Its ubiquitination signals tightly correlates with its Y817 phosphorylation pattern. Accordingly, the total level of TrkB is evidently reduced at 180 min, fitting with the previous findings [36–39]. 7,8-DHF treatment swiftly induces TrkB phosphorylation and the activation sustains for more than 3 h without inducing TrkB ubiquitination or degradation; by contrast, BDNF-triggered TrkB Y817 phosphorylation with a peak signal at 10 min, decreases at 60 min and fades away at 180 min. Clearly, these findings support that 7,8-DHF and BDNF activate TrkB with different mechanisms. It remains unclear how the activated TrkB signaling induced by 7,8-DHF is turned off eventually. Conceivably, 7,8-DHF and BDNF may induce differential to dephosphorylate the phosphorylated tyrosine residues of TrkB in the signalsomes within the cytoplasms.

## Structure-activity relationship (SAR) study of 7,8-DHF

Flavonoids are a large group of polyphenolic compounds containing a basic flavan nucleus with two aromatic rings (the A and the B rings) interconnected by a three-carbon-atom heterocyclic ring (the C ring). Flavonoids are divided into several big categories including flavone, flavonol, flavanone, flavanonol, flavans, anthocyanidins and isoflavonoids. 5,7-dihydroxylation is commonly found on the A ring, but 7-hydroxy ring is common in the isoflavonoids subgroups. The B ring generally has 4′-, 3′,4′- or a 3′,4′5′-hydroxylation pattern. Rare flavonoids like 7,8-DHF lack B-ring oxygenation. Flavonoids are the largest and the most diverse class of plant secondary metabolites. These compounds are naturally present in vegetables, fruits, and beverages. Bioflavonoids have been known for a long time to exert diverse biological effects. In particular, they are antioxidants and preventive agents against cancer [40]. Accumulating evidence suggests that flavonoids have the potential to improve human memory and neuro-cognitive performance via protecting the vulnerable neurons, enhancing neuronal function and stimulating neuronal regeneration [41]. Flavonoids also exert effects on LTP, one of the major mechanisms underlying learning,memory and cognitive performance through their interactions with the signaling pathways like PI 3-kinase/Akt [42], MAPK [43, 44] and PKC [45], etc.

The widespread distribution of flavonoids, their variety and their relatively low toxicity compared to other active plant compounds (e.g. alkaloids) mean that many animals, including humans, ingest significant quantities in their diet. Therefore, we tested a number of commercially available flavonoids for their TrkB agonistic activities. Our preliminary structural-activity relationship (SAR) study showed that the 7,8-dihydroxy groups are essential for the agonistic effect. We also found that the 8-hydroxy group in A ring is essential for the TrkB stimulatory effect, as 5,7-DHF, 5,6-DHF and 5,6,7-THF could not activate TrkB. The dihydroxy groups in B ring displays relatively higher TrkB agonistic activity when compared to compounds with dihydroxy groups in A ring. None of the single hydroxy flavone derivatives exhibited notable TrkB stimulatory effect and no trihydroxy flavones or dimethoxy flavones demonstrated any substantial effect [46].

The presence of 4′-position amino group enhances the agonistic effect of 7,8-DHF [46], which cannot be replaced by a hydroxy group. Interestingly, the 3′ position hydroxy group escalates 7,8-DHF’s agonistic activity and improves the hearing of animals thorough protecting spiral ganglion neurons from degeneration [46–48]. It is worth noting that the oxygen atom in C ring is also essential for the stimulatory activity as replacing this O atom with NH group abolishes the agonistic activity of 7,8-DHF. Together, our SAR study suggests that the catechol group (7,8-dihydroxy in A ring) might be indispensable for the agonistic activity and the 4′-hydroxy group on B ring reduces, whereas 3′-hydroxy group increases its activity.

To optimize the lead compound and conduct more comprehensive SAR studies, we synthesized dozens of 7,8-DHF derivatives via medicinal chemistry. The first generation of the optimized derivative is 4′-dimethylamino-7,8-DHF, which possesses higher agonistic effect on TrkB than the parental compound 7,8-DHF with longer in vivo activity. Because catechol group containing compounds usually possess poor pharmacokinetic profiles, as a part of our effort to optimize the lead compound 4′-dimethylamino-7,8-DHF, we synthesized numerous bioisoteres of this compound to enhance its biological or physical properties. Replacing the 7,8-dihydroxy groups with imidazole or urea ring in A-ring elevates the TrkB agonistic activity than the lead compound. Since the 4′-dimethylamino group is prone to demethylation during metabolism, we replaced the dimethylamino group with a pyrrolidino or mono-methylamino group to address this potential issue. Several rounds of organic synthesis of 7,8-DHF derivatives have been performed and we have obtained a couple of synthetic benzo-imidazole derivatives displaying EC50 of about 5–10 nM in primary neurons (unpublished data). Their in vivo pharmacokinetic (PK) profiles and oral bioavailability are now under investigation.

## Cytotoxicity of 7,8-DHF

7,8-DHF treatment did not induce any apparent toxicity in mice. In one of our studies, we compared the pathological changes induced by 7,8-DHF after feeding C57BL/6 mice at 5 mg/kg for 3 weeks. No adverse pathological change was detectable in the drug-treated kidney, liver, lung, muscle, spleen, cortex, hippocampus, heart, intestine and testis [46]. In addition, the complete blood count (CBC) analysis showed that there is no significant difference between drug-treated and the control saline-treated mice [49]. In another long-term feeding experiment, female mice receiving ~ 2.4 μg 7,8-DHF/day for 20 weeks also displayed normal CBC values [49]. These data support that 7,8-DHF is not toxic to the mice during the chronic treatment. Titration experiment also reveal that 7,8-DHF does not inhibit HEK293 cell proliferation at a dose up to 50 μM [46].

## Pharmacokinetics of 7,8-DHF

To study the 7,8-DHF’s PK profiles, we have performed a panel of in vitro ADMET assays. We found that 7,8-DHF is stable in liver microsomal assay but labile in hepatocytes, indicating that 7,8-DHF might be readily subjected to secondary modification-conjugation. Caco-2 permeability assay, parallel artificial membrane permeability assay (PAMPA) and PAMPA-BBB assays demonstrate that 7,8-DHF possess reasonable absorption rate and is able to penetrate BBB [50]. MDR1-MDCKII permeability assay also indicates that 7,8-DHF is a weak P-glycoprotein (Pgp) substrate (unpublished data). hERG assay reveals that 7,8-DHF exhibits IC_50_ >25 μM. CYP inhibition and induction assays reveal that 7,8-DHF has no time-dependent inhibition nor induction on major CYP enzymes. Concur with this result, 12 months chronic treatment or high dose acute treatment with 7,8-DHF suggests that 7,8-DHF is non-toxic to the rodents.

In vivo PK study shows that plasma 7,8-DHF concentration peaks at 10 min with 70 ng/ml, and brain 7,8-DHF also climaxes at 10 min with concentration of 50 ng/g of brain. 7,8-DHF in plasma can still be detected after 8 h (5 ng/ml) after administration. In contrast, only 7 ng/g of 7,8-DHF can be found in brain at 4 h and it is below the quantitative limit at 6 h [50]. In vivo metabolism study shows that 7,8-DHF is subjected to glucuronidation, sulfation and methylation [33]. Among these modifications, glucuronidation and sulfation are mainly responsible for the in vivo clearance of the flavonoids. Indeed, O-methylated metabolites including 7-methoxy-8-hydroxy-flavone (7M8H-flavone) and 7-hydroxy-8-methoxy-flavone (7H8M-flavone) can be detected in both the plasma and brain samples after oral administration of 7,8-DHF. Interestingly, these mono-methylated metabolites are still active in triggering TrkB activation in primary neurons and mouse brain [50].

Catechol containing compounds usually have short in vivo half-life and are prone to be cleared in the circulation system after oxidation, glucoronidation, sulfation or methylation. For instance, Apomorphine is a catechol-containing non-narcotic morphine derivative that acts as a potent dopaminergic agonist. Its metabolism occurs through several enzymatic pathways, including N-demethylation, sulfation, glucuronidation, and catechol-O-methyltransferase as well as by nonenzymatic oxidation [51]. L-DOPA is the mainstay of Parkinson’s disease (PD) therapy; this drug is usually administered orally, but it is extensively metabolized in the gastrointestinal tract, so that relatively little arrives in the bloodstream as intact L-DOPA. To minimize the conversion to dopamine outside the central nervous system, L-DOPA is usually given in combination with inhibitors of amino acid decarboxylase and COMT (catechol methyltransferase) [52]. Our preliminary in vivo PK study revealed that 7,8-DHF has t_1/2_ more than 2 h in mouse circulation after oral administration [50]. Conceivably, glucuronidation, sulfation and methylation pathways may explain the relative short half-life of 7,8-DHF and its synthetic derivatives.

To improve the poor PK profiles intrinsic to catechol-containing molecules, we synthesized numerous prodrugs by modifying 7,8-dihydroxy groups with esters, carbamates or phosphates to improve the oral bioavailability and brain exposure of 7,8-DHF. Currently, an optimal prodrug R7 has been found with favorable in vitro ADMET (absorption, distribution, metabolism, excretion and toxicity) characteristics. R7 exhibits approximately 18 % oral bioavailability with C_max_ of 1554.9 ng/ml, T_max_ of 0.28 h and T_1/2_ for PO of 2.32 h. Noticeably, 7,8-DHF plasma concentration released from R7 (PO, 50 mg/kg) is much higher than orally administrating the same dose of parent 7,8-DHF. The oral bioavailability is increased from 4.6 % (parental 7,8-DHF) to 84.2 % (R7). Accordingly, the brain exposure for 7,8-DHF is significantly increased by R7 than the parent compound upon oral administration of comparable dosage (unpublished data). TrkB and its downstream p-Akt/p-MAPK signalings are potently activated upon oral administration of R7, which is tightly correlating with 7,8-DHF concentrations in the animal brain. R7-provoked TrkB activation also fits well with the in vivo PK data, underscoring that the released 7,8-DHF from R7 prodrug triggers a long-lasting TrkB signalings in the mouse brain. This prodrug is now under preclinical IND-enabling study for the indication of Alzheimer’s disease.

### 7,8-DHF displays robust therapeutic efficacy toward Alzheimer’s disease

There is mounting evidence that 7,8-DHF mimics the physiological activities of BDNF and exhibits promising therapeutic efficacy toward various neurological diseases including Parkinson’s disease (PD) [26], Huntington’s disease (HD) [27], ALS (Amyotrophic lateral sclerosis) [53, 54], Alzheimer’s disease (AD) [55–58], Posttraumatic Stress Disorder (PTSD) [59], and Rett Syndrome [60]. Moreover, 7,8-DHF displays therapeutic effect toward axon regeneration [61], and spiral ganglion degeneration [48]. Noticeably, it also demonstrates therapeutic activities in mental diseases like depression [33, 56, 62, 63]. Here, we focus on discussing its effects in treating AD, the leading cause of dementia worldwide, which is characterized by the accumulation of the β-amyloid peptide (Aβ) within the brain along with deposition of hyperphosphorylated and cleaved microtubule-associated protein Tau. It is suggested that reductions of BDNF content or TrkB inactivation may play a role in the pathogenesis of AD. Indeed, BDNF expression is reduced in the brain of AD patients and delivery of *BDNF* gene has been shown as a novel potential therapeutic in diverse models related to AD [64]. BDNF also displays a protective role against AD pathogenesis by increasing learning and memory of demented animals [65]. Thus, these preclinical evidence strongly supports that BDNF might be useful as a therapeutic agent for treating AD.

Reduced acetylcholine neurotransmission due to loss of neurons in the basal forebrain and depletion of choline acetyltransferase are observed in AD pathology. Currently, there are two types of medication to treat AD: cholinesterase inhibitors and NMDA antagonist. However, these drugs can only delay the inevitable symptomatic progression of the disease without eliminating the main neuropathological hallmarks of the disease (i.e. formation of senile plaques and neurofibrillary tangles) nor rescuing the neuronal loss. 7,8-DHF potently stimulates hippocampal progenitor neurogenesis. For instance, oral administration of 7,8-DHF (5 mg/kg) in wild-type C57BL/6 J mice for a few weeks strongly induces neurogenesis [46]. Intraperitoneal administration of 7,8-DHF also elicits robust neurogenesis in depressive vulnerable or non-vulnerable rat [62]. This neurotrophic effect by 7,8-DHF has also been observed in APP/PS1 AD mouse model [66]. Devi and Ohno showed that 7,8-DHF rescued memory deficits in transgenic mice that co-express five familial Alzheimer’s disease mutations (5XFAD) during the spontaneous alternation Y-maze task. In addition, 7,8-DHF restores deficient TrkB signaling in 5XFAD mice without affecting endogenous BDNF levels. While 5XFAD mice exhibit elevations in the β-secretase enzyme (BACE1) that initiates amyloid-β (Aβ) generation, as observed in sporadic AD, 7,8-DHF blocks BACE1 elevations and lowers the levels of the β-secretase-cleaved C-terminal fragment of amyloid precursor protein (C99), Aβ40, and Aβ42 in the brains of these mice. Most strikingly, they demonstrated that BACE1 expression can be decreased by 7,8-DHF administration in wild-type mice, suggesting that BDNF-TrkB signaling is also important for downregulating baseline levels of BACE1. Hence, this study supports that TrkB activation with systemic 7,8-DHF administration can ameliorate AD-associated memory deficits, attributable to reductions in BACE1 expression and β-amyloidogenesis [55]. Nevertheless, the authors employed a subchronic paradigm (10 days intraperitonial injection) in aged 5X FAD mice (12–15 months old mice). Since 5X FAD mice develop amyloid plaques at 2 months old and exhibit cognitive defects at 5 months of age, we employed a different treatment strategy: feeding the mice at 2-months-old till 5-months-old and monitored the cognitive activity in Morris Water maze. In addition, we examined the amyloid plaque deposit, synapse formation and long-term potentiation (LTP) at the end of the treatment. Our data showed that 7,8-DHF protects primary neurons from Aβ-induced cell death and promotes dendrite branching and synaptogenesis. Chronic oral administration of 7,8-DHF activates TrkB signaling and prevents Aβ deposition in 5XFAD mice [56]. In alignment with these findings, 7,8-DHF significantly increases spine density and reduces synaptic and neuronal loss in Cam/Tet-DTA, an inducible model of severe neuronal loss in hippocampus and cortex, and demonstrates substantial improvements in spatial memory in the lesioned mice [58]. These results strongly suggest that 7,8-DHF represents a novel oral bioactive therapeutic agent for treating AD.

### 7,8-DHF inhibits obesity through activating muscular TrkB

Obesity is a metabolic disorder with increasing prevalence worldwide. According to the World Health Organization (WHO), more than 39 % (~1.9 billion) of adults are overweight. Of these, over 600 million (~13 %) are obese in 2014. These numbers have been doubled since 1980. Therefore, developing effective pharmacotherapy to control excess body weight gain is a hot research direction nowadays.

In addition to the neurotrophic activities, BDNF/TrkB signaling also plays a critical role in food intake and body weight control. In rodents, pharmacological treatments with BDNF induce a reduction of food intake, whereas genetic models with reduced BDNF/TrkB signaling display hyperphagia and obesity [67, 68]. Recent evidence indicates that BDNF acts as an energy metabolism regulator in both CNS and peripheral organs. It has been reported that BDNF levels are low in obesity or patients with type 2 diabetes [68, 69]. BDNF is expressed in non-neurogenic tissues, including skeletal muscle, and exercise increases BDNF levels in brain, plasma and skeletal muscle. Pederson et al. reported that BDNF increased phosphorylation of AMP-activated protein kinase (AMPK) and acetyl coenzyme A carboxylase (ACC) and enhanced fatty oxidation both in vitro and *ex vivo*. These data points to the fact that BDNF is a contraction-inducible protein in skeletal muscle that is capable of enhancing lipid oxidation via activation of AMPK. Thus, BDNF appears to be an active player in both neurobiology and peripheral metabolism [70].

Because BDNF has anti-obesity activity by suppressing food intake, we thus initiated a test to see if 7,8-DHF can be used to prevent the development of obesity. We investigated the effect of 7,8-DHF (drinking the dissolved 7,8-DHF in water) on mouse body weight gain under chow diet or high-fat diet (HFD) feeding for 6 months [49]. To our surprise, 7,8-DHF consumption does not suppress food intake, which is in contrast to BDNF administration. Nevertheless, we found that 7,8-DHF significantly decreases the body weight gain in both chow diet and HFD paradigms, with more striking effect on HFD. The white adipocyte tissue (WAT) mass is significantly decreased about 20–30 % in 7,8-DHF-treated HFD group. Indirect calorimetry study showed that 7,8-DHF treatment decreases RER (respiratory exchange ratio), favoring the usage of lipid as the main fuel. Our study also reveals the mechanism of the anti-obesity actions by 7,8-DHF. By performing experiments in animal model and cell culture (C2C12) system, we identified that 7,8-DHF mainly acts on the muscle TrkB receptors to induce uncoupling protein 1 (UCP1) expression and activates AMP-activated protein kinase (AMPK). As a result, the energy expenditure and lipid oxidation in 7,8-DHF activated muscle cells are increased, leading to the lean phenotype observed. Unexpectedly, this anti-obesity effect is predominantly associated with female mice but not male mice, presumably due to estrogen content of the animals. Mice with 7,8-DHF treatment also exhibit improved blood insulin concentration, lower blood glucose level and increased insulin sensitivity in tissues such as liver, fat and muscles, suggesting 7,8-DHF is effective in alleviating the obesity-induced diabetes as well. These exciting findings identify a new function of BDNF/TrkB signaling in the skeletal muscle that the cascade controls cellular energy expenditure, which also provides the pre-clinical evidence that 7,8-DHF administration is an effective means to suppress body weight gain during energy surplus.

## Perspectives and future directions

7,8-DHF is a broadly validated small molecule that imitates the biological functions of BDNF via directly binding to the TrkB ECD to trigger TrkB receptor dimerization and autophosphorylation [26]. It simulates the physiological functions of BDNF like promoting neuronal survival, elevating synaptogenesis and LTP on aged hippocampal slides and enhancement of learning and memory [26, 71, 72]. More important, 7,8-DHF displays a robust therapeutic efficacy in numerous neurological and metabolic diseases. However, it should be noted that the dosage of 7,8-DHF in clinical application has to be determined carefully as over-activation of the TrkB signaling may result in devastating consequences. In transgenic mice that overexpress BDNF in the forebrain, the animals exhibit spatial learning deficits at 2–3 months of age, followed by the emergence of spontaneous seizures at ~6 months, which is possibility a result of neuronal hyperexciation [73–75]. Because BDNF acts at central synapses in pain pathways both at spinal and supraspinal levels, prolonged TrkB activation may also interfere with the nociception pathway, leading to pain hypersensitivity [76].

Further investigations on the biochemical and mechanistic natures of 7,8-DHF are also necessary to boost its applications. For instance, the precise molecular details of how 7,8-DHF induces TrkB dimerization and activation are still unclear. For instance, how could 7,8-DHF mimic so many aspects of the physiological activities of the macromolecule BDNF, given that 7,8-DHF is only 254 dalton, which is 1 % of polypeptide hormone BDNF in size? Further, the tyrosine phosphorylated residues on TrkB intracellular domain and their relative abundance are different after BDNF and 7,8-DHF stimulations [32]. What downstream effects may these molecular differences incur in view that although both PI3K/Akt and MAPK downstream pathways are activated? Clearly, further biochemical investigations into these aspects are undoubtedly needed. Additional medicinal chemistry work is also required for optimizing this promising lead compound into a nanomolar binding affinity small molecular clinical candidate, which will not only provide an innovative pharmacological intervention for treating various human disorders but also present a useful tool for dissecting the biological actions of BDNF/TrkB signalings. For example, the availability of a TrkB PET tracer, which does not exist currently, would accelerate TrkB therapeutic development and provide insight into the role of TrkB and its expression levels in neuropsychiatric and neurodegenerative diseases, as well monitoring the change of TrkB levels during antidepressant or therapeutic neuro-regeneration treatments.

## Conclusions

7,8-dihydroxyflavone is a promising small molecular BDNF mimetic compound, which fully mimics the physiological actions of BDNF from neuronal survival, synpatogenesis, axonal regeneration etc. Notably, it is orally bioactive and is safe for chronic treatment. It has been extensively validated in various BDNFimplicated disease models. Hence, it acts as a good lead compound for further medicinal modification for optimizing its therapeutic efficacy toward various BDNF-mediated human disorders.
